# Craig plot 2.0: an interactive navigation in the substituent bioisosteric space

**DOI:** 10.1186/s13321-020-0412-1

**Published:** 2020-01-28

**Authors:** Peter Ertl

**Affiliations:** Novartis Institutes for BioMedical Research, CH-4056 Basel, Switzerland

**Keywords:** Craig plot, Bioisosteric design, Visualisation, Chemical space, Hammett sigma constant, Hansch–Fujita pi parameter

## Abstract

Bioisosteric replacement is a standard technique that is used in medicinal chemistry to design analogs of bioactive molecules with similar biological activity and with additional improved characteristics. Successful application of this technique relies on a good knowledge of physicochemical properties of common organic substituents and an efficient way to navigate their space. In this study the properties of the most common substituents present in bioactive molecules are analysed and a freely-available web tool https://bit.ly/craigplot that allows visualization, analysis and selection of bioisosteric substituents is presented.
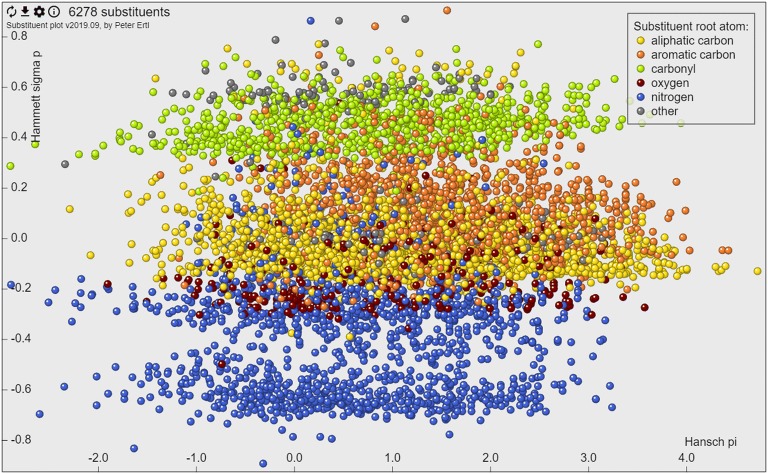

## Introduction

A bioisosteric replacement may be defined as the replacement of a part of a bioactive molecule with a substructure that is similar in size and exhibits similar physicochemical properties. Bioisosteric transformations are used in the process of lead optimization to improve the properties of potential drug candidates, such as bioavailability, selectivity or transport characteristics, or to remove unwanted side effects such as toxicity or metabolic liabilities while also endeavouring to maintain the original bioactivity of the molecule [1]. Bioisosteric replacements are also used in situations where the optimization is intended to improve the synthetic accessibility of the molecule. In the earlier project stages usually the central molecule scaffold is modified, while in the later stages substituents attached to the scaffold are optimised. Classical textbook examples of substituents that are bioisosterically equivalent include phenyl with thiophenyl, and carboxylic acid with tetrazole. Identifying bioisosteric analogues of more complex substituents, however, is not so trivial. This requires a considerable amount of medicinal chemistry experience. Even if this experience is available, the identification of a bioisosterically suitable group with an optimal balance of steric, hydrophobic, electronic and hydrogen-bonding properties, all of which influence ligand-receptor interactions, usually requires an intensive procedure of trial and error.

Another field where the good knowledge of substituent property space is required is combinatorial library design. Based on the selected design strategy one has to identify set of representative, diverse or focused building blocks. The methods used to characterize these building blocks are similar to those used for bioisosteric design. In the former case, however, the basic requirement is to cover the desired property space as broadly as possible while, in the later case, selected isosteres should be similar in properties.

Various computational methods can provide useful help in navigating the space of substituents and identifying the groups with similar physicochemical properties. One of the first rational approaches to navigate this space was introduced by Craig. He suggested a two-dimensional plot (called now Craig plot) where Hammett *σ* and Hansch *π* substituent constants were displayed for a set of substituents [2]. This plot allows the selection of substituents with diverse properties—substituents should be selected in this case from different quadrants, but it also helps to select groups that are close together on the assumption that they will have similar effects on bioactivity. The Craig plot was quite simple but efficient approach to the identification of bioisosteric substituents. Another classical medicinal chemistry technique used to navigate the substituent property space was introduced by Topliss [3]. He suggested a substituent decision tree that should guide a medicinal chemist to the most potent analogue by rational analysis of the activity order observed so far. His classical approach was recently revisited and enhanced by applying modern cheminformatics techniques to processing the substituents and large amount of related bioactivity data extracted from the ChEMBL database [4]. Many other approaches to navigate the substituent property space to help medicinal chemists have been explored, see [5] for a review. In the present study we contribute to this field by analyzing a large collection of substituents extracted from bioactive molecules and introducing a web-based interactive tool that allows interested scientists to navigate the substituent space and select bioisosteric substituents.

## Implementation

### Selection of the substituents

The substituents analysed in this study were extracted from the bioactive molecules in the ChEMBL database [6]. Molecules with activity below 10 µM on any target were considered to be “bioactive” (altogether ~ 700,000 molecules). These molecules were fragmented in a way described in [7]. Basically all chemically activated (breakable) bonds were cut. This included bonds between ring and nonring atoms, between carbons and heteroatoms and bonds adjacent to multiple bonds (mostly carbonyls). Substituents with up to 12 heavy (non-hydrogen) atoms were collected. This procedure provided a database of 143,180 substituents. The most common substituents, i.e. those present in 50 or more molecules, altogether 6278, were then used as a basis for the development of the interactive tool. More detailed cheminformatics analysis of all the substituents extracted from ChEMBL is provided in the “Results” section.

### Calculation of substituent properties

The substituents were characterized by the two important properties—namely the Hammett *σ* constant and the Hansch–Fujita *π* parameter. The *σ* constant characterizes the electron-donating power of substituents [8] while the *π* parameter describes their hydrophobicity, defined as the difference between the octanol–water partition coefficient (log*P*) of a substituted molecule against its unsubstituted parent [9]. These two properties were used to characterise substituents also in the original Craig’s paper.

A significant issue in using the experimental data to characterise the substituents, however, is the scarceness of this information. In our earlier study we find out that only 63 of the 100 most common substituents have been characterized experimentally [10]. Therefore one has to rely on reliable and robust predictive models that allow calculation of substituent properties in silico. In this study the *π* hydrophobicity substituent parameters were calculated by the well-known method of Ghose, Crippen and Wildman that is based on atom contributions [11]. The acceptor and donor power of substituents was characterised by a parameter compatible with the Hammett *σ* constant calculated from atomic charges of substituted benzene derivatives containing the respective substituents. Details of this method are provided in our earlier study [10].

The calculated substituent parameters agree well with the experimental values. For the 200 most common substituents identified in this study the experimental *π* values are available for 86 [9]. The experimental and in silico values correlates with *r*^*2*^ = 0.90. For Hammett *σ* para constants the data are available for 83 substituents [8] and the correlation is *r*^*2*^ = 0.89. In this study the Hammett *σ* para and not the *σ* meta values are used, since their span is larger (for example, the difference between the nitro and dimethylamino groups is 0.97 for *σ* meta and 1.52 for *σ* para) and therefore provides better separation of substituents.

The data set of calculated *π* and *σ* constants for the 6278 common organic substituents used in this study may be downloaded from the related GitHub repository (see the availability section).

### Web tool

**Fig. 1:**
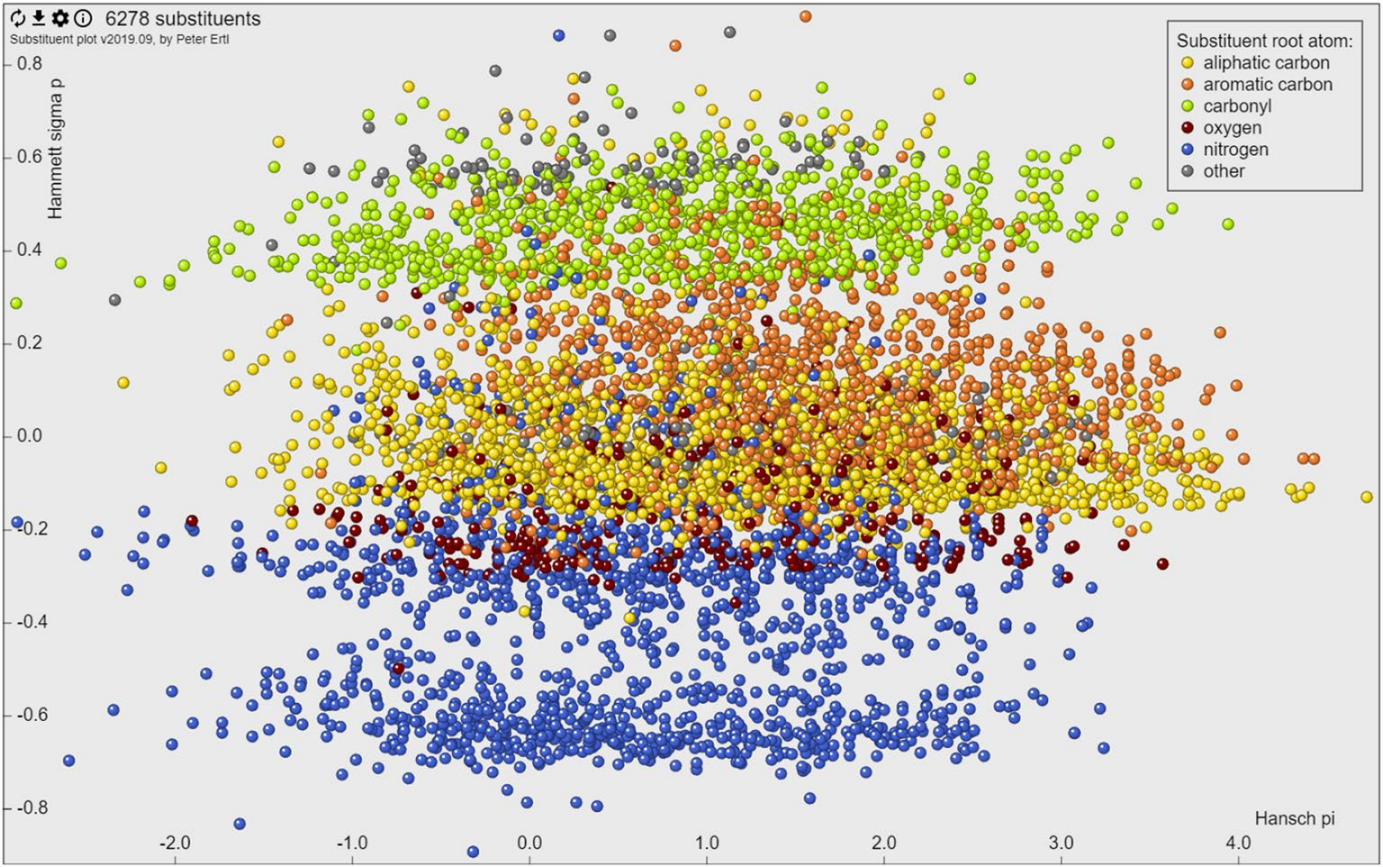
The web interface

**Fig. 2:**
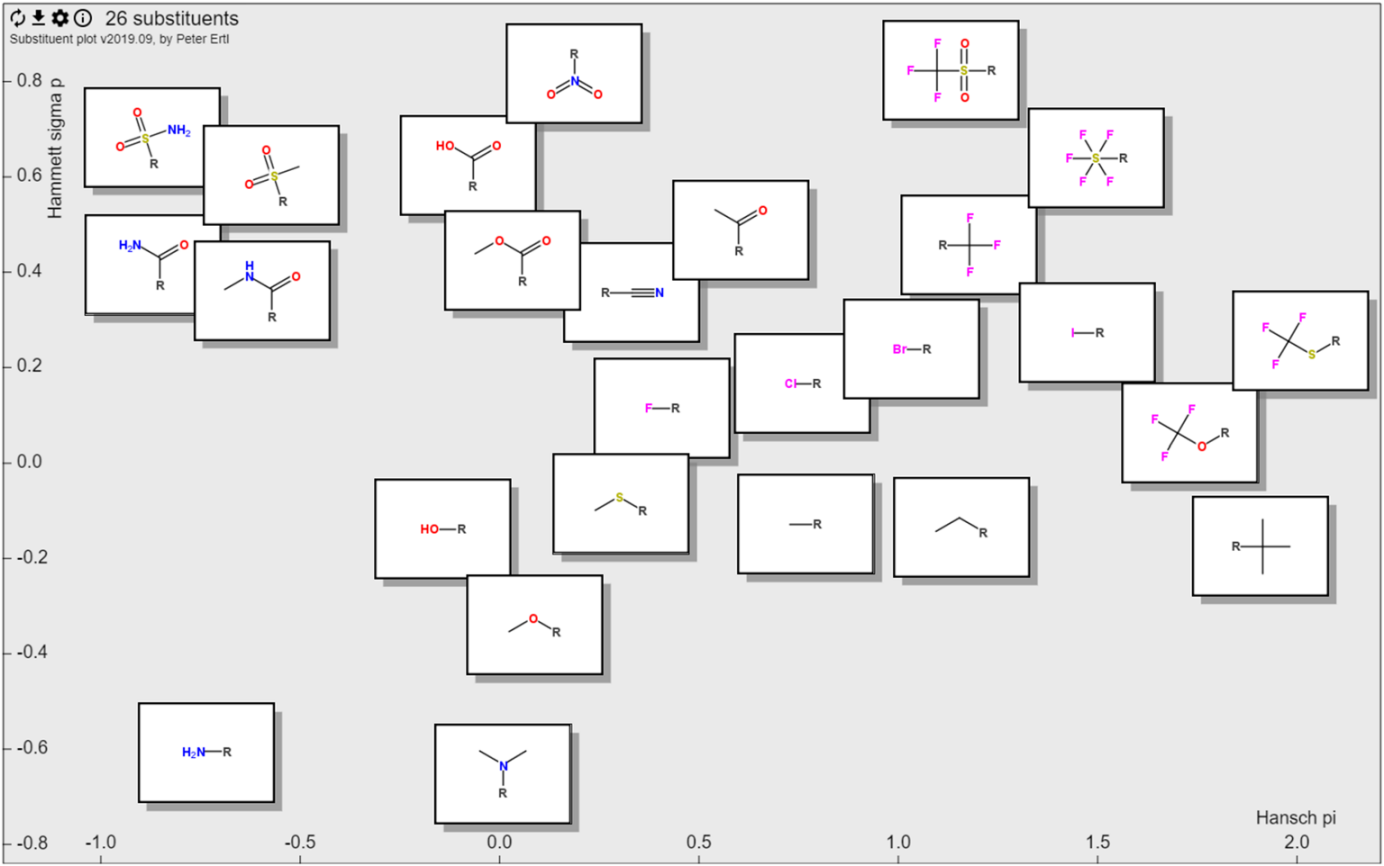
Craig plot 2.0—visualization of logP and Hammett constants of the 26 substituents used in the original Craig paper [2]

The web tool that allows interactive navigation in the substituent property space was written in JavaScript using the JQuery framework [12]. The actual graphics is rendered using the canvas HTML5 element (Fig. 1). The interface allows to visualize and select substituents, “glue” the molecule image to the point representing the substituent or show images for all displayed groups. This option enables for example visualization of the Craig plot for the 26 substituents discussed in the original Craig’s paper [2] (Fig. 2). The selected substituents may be downloaded as SMILES, including their calculated properties. Selection of part of the plot and zooming to it allows focusing on a particular portion of the property space. An option menu allows selection of various subsets of substituents based on their type (characterised by the root atom of substituent), connection point (type of atom in the molecule to which the substituent is connected), substituent size or substituent frequency. Integrated help provides information about the use of the tool as well as about the keyboard shortcuts that make the work with the tool more efficient.

## Results

**Fig. 3:**
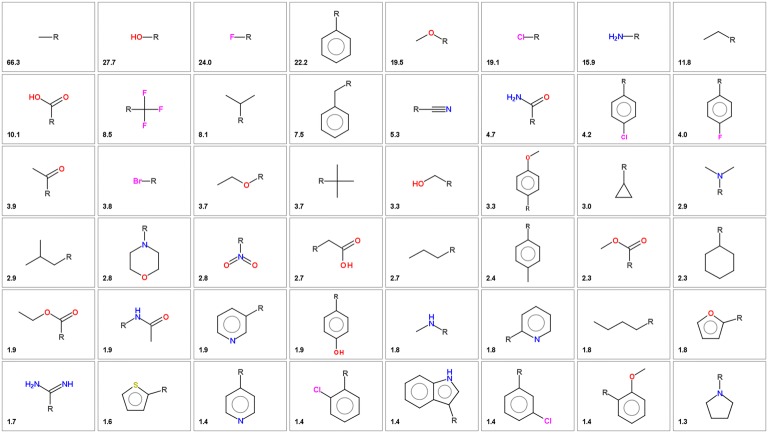
The most common substituents extracted from bioactive molecules in ChEMBL. The number indicates the percentage of molecules having this substituent

As mentioned in the “Implementation” section, fragmentation of ~ 700,000 bioactive molecules from ChEMBL generated 143,180 substituents with up to 12 non-hydrogen atoms. The most frequent substituents are shown in Fig. 3. The distribution of substituents shows a typical power law (or “long tail”) distribution with few common substituents and a large number of infrequent substituents. Only 67 substituents are present in more than 1% of the molecules, 586 in more than 0.1% of the molecules, 70,895 substituents (49.5%) are singletons (present only in one molecule).

The substituents can be classified based on their root atom, i.e. the atom that is connected to the main skeleton. For more detailed classification also atom environment farther from the root atom may be considered. Results of such classification are shown in Table 1. Another way to classify substituents is to group them according to the type of atom they are connected to—for example substituents connected to nitrogen, carbonyl carbon or aromatic carbon. Results of this classification are shown in the last column of Table 1. Both of these classification types are important from the medicinal chemistry point of view, since they allow chemists to select the building blocks best suited for a particular environment and required reaction type.

**Table 1:** Substituents clustered according to their root atoms

R	%	R—level2	%	Attached to (%), the * represents any extension
R–C	48.1	R–C–C	9.0	N, n (36), C (29), O (16), c (10), X (4), C(=O)* (4)
		R–C(–C)–C	4.9	C (39), N, n (37), c (11), O (8), C(=O)* (5), X (1)
		R–C(=O)–N	3.9	C (50), c(34), N, n (13), O (2), C(=O)* (1)
		R–C(=O)–C	3.7	N, n (76), c (9), O (9), C (6)
		R–C–c	2.9	N, n (42), C (34), O (10), c (5), C(=O)* (4), X (4)
		R–C–N	2.8	c (52), C (33), C(=O)* 14
		R–C(–C)–N	2.4	C(=O)* (53), C (32), c (15)
		R–C(=O)-c	2.0	N, n (74), C (12), O (7), c (7)
		R–C(–C)-O	1.7	C (69), c (12), N, n (9), C(=O)* (6), O (4)
		R–C =C	1.4	c (43), C(=O)* (40), C (12), N, n (3), X (2)
		R–C(–C)–c	1.2	N, n (71), C (11), c (6), O (6), C(=O)* (5)
		R–C–C–O	1.0	C (64), c (20). C(=O)* (14)
R–c	20.6	R–c(:c):c	10.9	C (42), N, n (21), c (18), C(=O)* (8) O (6), X (5)
		R–c(:n):c	5.2	C (30), N, n (28), c (22), C(=O)* (13), O (4), X (3)
		R–c(:n):n	1.9	c (31), C (25), N, n (24), X (15), C(=O)* (4), O (2)
R–N	19.6	R–N–C	8.7	C(=O)* (34), C (34), c (32)
		R–N(–C)–C	4.7	C (42), c (33), C(=O)* (25)
		R-N–c	3.1	C(=O)* (58), c (28), C (13)
R–O	6.1	R–O–C	4.0	c (70), C(=O)* (17), C (14)
		R–O–c	1.8	C (54), c (41), C(=O)* (5)
R–S	3.2	R–S–C	1.0	c (69), C (30)
R–n	2.0	R–n(:c):c	1.1	C (71), c (28), C(=O)* (1)

The substituent substructures are shown in SMILES-like notation (atoms in uppercase are aliphatic, in lowercase aromatic, colon is an aromatic bond and the comma between atomic symbols a logical OR). Only substituent classes with more than 1% frequency are shown

The information in the Table 1 was obtained by processing all extracted substituents, not only the unique ones (that means that various substituent types contributed as many times as they are present in ChEMBL, not only once). Altogether information about more than 6 million substituents were processed to generate this table.

More than two-thirds of all substituents are connected to the molecule through carbon (48.1% aliphatic and 20.6% aromatic), followed by aliphatic nitrogen (19.6%), oxygen (6.1%), sulfur (3.2%) and aromatic nitrogen (2%). When considering also the second connection level the largest group are phenyl (and possibly its heterocyclic analog) derivatives (10.9%), aliphatic alkyls (9%) and alkylamino groups (8.7%). Various carbonyl substituents (amides, esters and ketones) form together 9.6% of the all substituents.

According to the author’s knowledge this type of information about the classification of bioactive substituents according to their types, as well as about the preferred attachment points is not available in the scientific literature so far. Such information may be used not only in medicinal chemistry applications to select appropriate set of bioisosteric analogs or building blocks for combinatorial library design, but also in other cheminformatics workflows. With the current boom of various in silico molecule generation methods the detailed information about the substructure properties of substitution patterns would allow for example to validate and also fine tune the molecule generators to represent well the existing chemical space of bioactive molecules.

## Conclusions

The interactive web tool presented here allows chemists to navigate the chemical space of the common bioactive substituents. Using its sophisticated query features the users are able to answer the questions that were not in an easy reach of medicinal chemists so far, for example.

- Show me the strongest donors with up to 8 atoms that are not too hydrophobic
- Show me common aromatic rings that are preferably connected to other aromatic system
- Show me the most common substituents attached through oxygen
- Show me the most hydrophilic substituted aromatic rings.

The identified subset of substituents may be downloaded or further refined by manual selection.

Additionally, the whole database of over 6000 bioactive substituents with calculated properties that can be used to support various cheminformatics activities like bioisosteric design, combinatorial library design or diversity selection may be downloaded.

### Availability and requirements

- Project name: Craig Plot 2.0
- Project home page: https://peter-ertl.com/molecular/substituents/craigplot.html
- GitHub: https://github.com/peter-ertl/craigplot
- Operating system: Web tool—platform independent
- Programming language: JavaScript
- Other requirements: none
- License: BSD 3-clause
- Any restrictions to use by non-academics: no.

## Data Availability

The web application described in this article is freely available at https://bit.ly/craigplot. The list of 6278 substituents as SMILES strings together with calculated Hansch–Fujita *π* hydrophobicity constants and Hammett para *σ* constants may be downloaded from the project GitHub repository.
